# Synthetic photoplethysmogram generation using two Gaussian functions

**DOI:** 10.1038/s41598-020-69076-x

**Published:** 2020-08-17

**Authors:** Qunfeng Tang, Zhencheng Chen, Rabab Ward, Mohamed Elgendi

**Affiliations:** 1 Department of Electrical and Computer Engineering, University of British Columbia, Vancouver, BC Canada; 2 School of Electronic Engineering and Automation, Guilin University of Electronic Technology, Guilin, China; 3 Faculty of Medicine, University of British Columbia, Vancouver, Canada; 4 BC Children’s and Women’s Hospital, Vancouver, Canada

**Keywords:** Cardiovascular diseases, Diagnostic markers, Biomedical engineering, Electrical and electronic engineering

## Abstract

Evaluating the performance of photoplethysmogram (PPG) event detection algorithms requires a large number of PPG signals with different noise levels and sampling frequencies. As publicly available PPG databases provide few options, artificially constructed PPG signals can also be used to facilitate this evaluation. Here, we propose a dynamic model to synthesize PPG over specified time durations and sampling frequencies. In this model, a single pulse was simulated by two Gaussian functions. Additionally, the beat-to-beat intervals were simulated using a normal distribution with a specific mean value and a specific standard deviation value. To add periodicity and to generate a complete signal, the circular motion principle was used. We synthesized three classes of pulses by emulating three different templates: excellent (systolic and diastolic waves are salient), acceptable (systolic and diastolic waves are not salient), and unfit (systolic and diastolic waves are noisy). The optimized model fitting of the Gaussian functions to the templates yielded 0.99, 0.98, and 0.85 correlations between the template and synthetic pulses for the excellent, acceptable, and unfit classes, respectively, with mean square errors of 0.001, 0.003, and 0.017, respectively. By comparing the heart rate variability of real PPG and randomly synthesized PPG for 5 min in 116 records from the MIMIC III database, strong correlations were found in SDNN, RMSSD, LF, HF, SD1, and SD2 (0.99, 0.89, 0.84, 0.89, 0.90 and 0.95, respectively).

## Introduction

Cardiovascular disease (CVD) is the leading cause of death and morbidity around the world^1^. The number of patients suffering from CVD increases each year. An electrocardiogram (ECG) is the most widely used method for detecting CVD; however, photoplethysmography (PPG) has increasingly been used to measure cardiovascular status over the last decade. An increasing number of PPG-based devices, such as smartphones and wearable devices, are now being used for monitoring and primary health screening^2,3^. PPG detects blood volume changes in the microvascular tissue bed caused by the pressure of circulating blood.

PPG is usually measured at body extremities, such as fingers, ears, and wrists^4^. It provides valuable physiological and pathological information about the cardiovascular system. The PPGs waveform characteristics and harmonic information can be related to the changes in the characteristic parameters of the cardiovascular system. Applications of PPG sequences include assessment of heart rate, oxygen saturation^6^, blood pressure^7^, cardiac output^8^, respiration rate^5^, and other indicators of cardiovascular function. The shape of the PPG waveform is variable; it differs from subject to subject and depends upon the measurement location and an individual’s health status.

**Figure 1:**
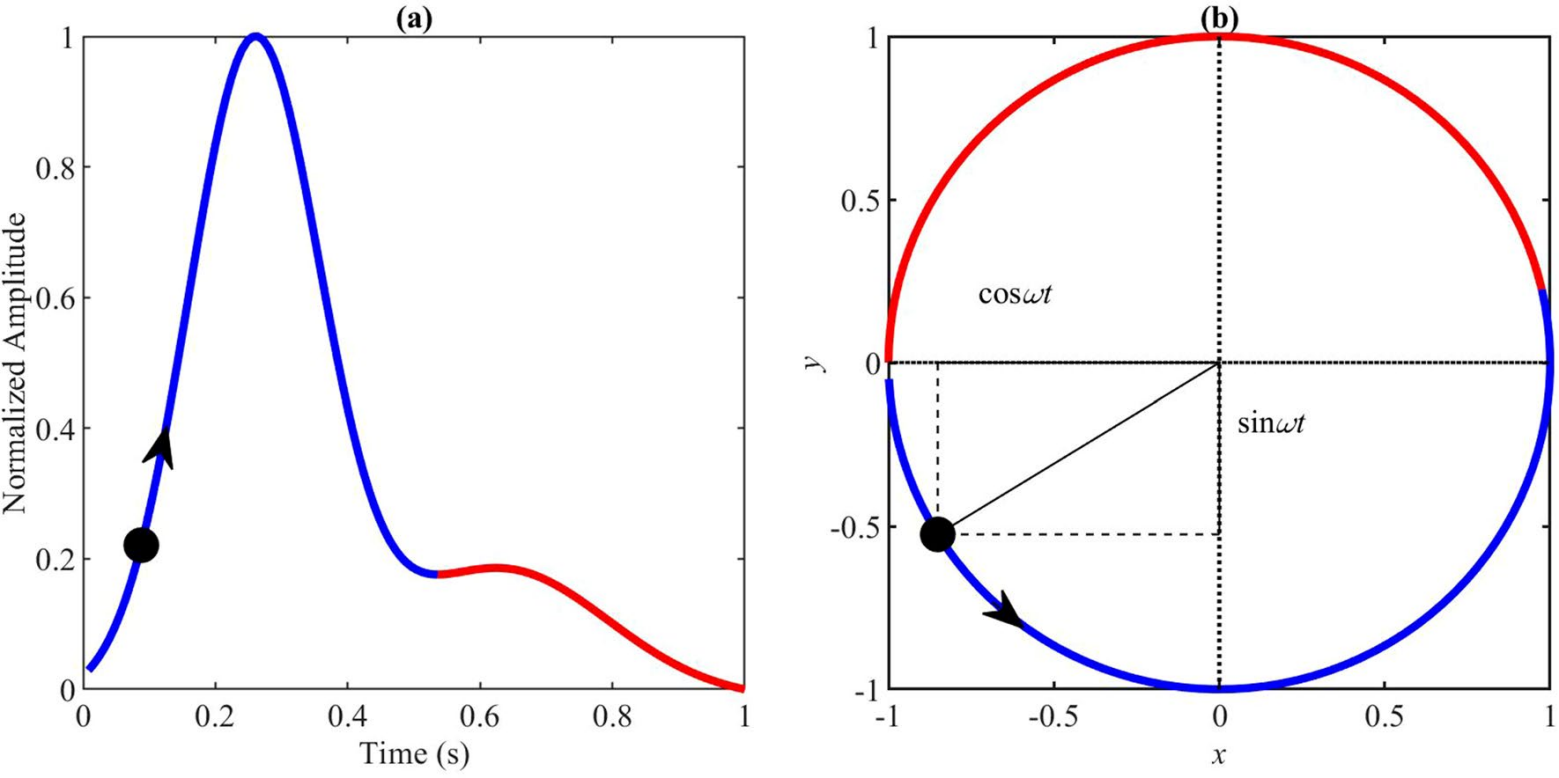
The main idea of the proposed modeling method. The blue color represents the systolic wave, and the red color represents the diastolic wave. (**a**) One-beat PPG waveform. (**b**) The circular motion principle. $\omega$ is the angular velocity; it is fixed per pulse, but changes with different pulses. This figure shows how the circular motion will be used to generate a PPG waveform in a periodical manner.

The PPG waveform represents one cardiac cycle, comprising the onset, the systolic peak, the diastolic peak, and the dicrotic notch^9^. The onset is the starting point of the PPG beat. It corresponds to the point at which the heart pumps blood to the blood vessels. It is also the endpoint of the previous beat. The systolic wave, shown in blue in Fig. 1a, is the segment from onset to the dicrotic notch, caused by the rapid pumping of blood from the left ventricle, a rapid rise in arterial blood pressure, and the expansion of the arterial wall. Subsequently, at the late stage of left ventricular pumping, the blood flow velocity slows down, the blood flow into the aorta is less than that to the periphery, the dilated artery begins to shrink back, and the arterial blood pressure gradually decreases.

The diastolic wave, shown in red in Fig. 1a, is the point from the dicrotic notch to the endpoint. The left ventricle diastolic reflux pushes the aortic valve closed, and arterial blood pressure continues to drop. The dicrotic notch is associated with the closing of the aortic valve^10^. Note that a number of factors, such as gender, age, and disease, can change the morphology and duration of the PPG pulse, making it appear different from a normal PPG pulse.

There are four publicly available PPG databases: Multiparameter Intelligent Monitoring in Intensive Care (MIMIC III)^11^, BioSec.Lab^12^, PPG-BP^13^, and Wrist PPG^14^. The MIMIC database signals are recorded at a sampling rate of 125 Hz and have different time durations, but the subject’s condition at the time of measuring is not clear. The signals were recorded at 100 Hz in the BioSec.Lab PPG dataset with a 3-min duration of each segment, and these subjects are measured at the fingertip, and in different conditions, such as relax, after exercise, short time-lapse and long time-lapse. The signals recorded in the PPG-BP database are sampled at a rate of 1 kHz for 2.1 s, and the measuring site is the fingertip. In the Wrist PPG database, the signals were recorded at a sampling rate of 256 Hz and varying durations, and recorded during exercise, the measuring site is the wrist. Typically, algorithms developed and tested on these databases are database-dependent. To evaluate developed algorithms reliably, it needs to test them in different conditions. Although the recording conditions of different databases may differ, there are few options for different conditions, which makes it challenging to evaluate the performance of currently available algorithms in different clinical settings over a range of noise levels and sampling rates. There is a need for a synthesizer that can generate PPG dynamically (with variable lengths, different sampling rates, different types of morphologies, and noise levels). Besides, synthesized PPG signals can also be used to reconstruct missing segments of the PPG signal.

Modeling of PPG pulses has been attempted earlier studies. Shariati and Zahedi compared four linear parametric models^15^. Wang et al. used multi-Gaussian functions to fit a single PPG waveform^16^. They compared the simulation effects of different numbers of Gaussian functions. However, these only addressed a single heartbeat. Other papers generated a PPG signal from a sequence of pulses. Martin-Martinez et al.^17^ proposed stochastic modeling to synthesize PPG signals. They designed a single-pulse model based on two Gaussian functions comprising 10 parameters. The mean of two autoregressive moving average models was used to approximate these 10 parameters; however, the original PPG signal is required for the time evolution of the 10 parameters in the model. Sološenko et al. proposed a model for simulating PPG during atrial fibrillation^18^. They used one log-normal and two Gaussian waveforms to simulate a single pulse and extract the RR intervals from the ECG in order to connect individual PPG pulses according to the RR intervals; however, their model required an ECG signal as an input parameter. In contrast, this paper proposes a dynamic model that can generate synthetic PPGs with different lengths and sampling frequencies.

## Results

The dataset we used was 116 subjects from the MIMIC III database^11^. The PPG for each subject was recorded at a sampling frequency of 125 Hz for a duration of 5 min.

### Similarity in single pulses

To test the ability of the model to simulate different shapes of PPG pulse, the model was used to emulate three classes^19^ of normalized PPG templates (referred to as excellent PPG, acceptable PPG, and unfit PPG). Excellent PPG occurs when the systolic and diastolic waves are salient. Acceptable PPG occurs when the systolic and diastolic waves are not salient, but the heart rate can be determined. Unfit PPG occurs when the heart rate cannot be determined and the systolic and diastolic waves cannot be distinguished. There is one template for each class of PPG pulse. These templates are extracted manually from the database we used and from different subjects.

The mean square error (MSE) and the correlation coefficient were used to evaluate the model-fitting accuracy of the algorithm. MSE is expressed as follows:

1 $$\begin{aligned} MSE = \frac{1}{l}\sum _{n=1}^l{(s(n) - z_p(n) )^2}, \end{aligned}$$

where $z_p(n)$ and $s(n)$ represent individual points of the synthetic PPG and real PPG, respectively, and $l$ is the length of the signal.

**Figure 2:**
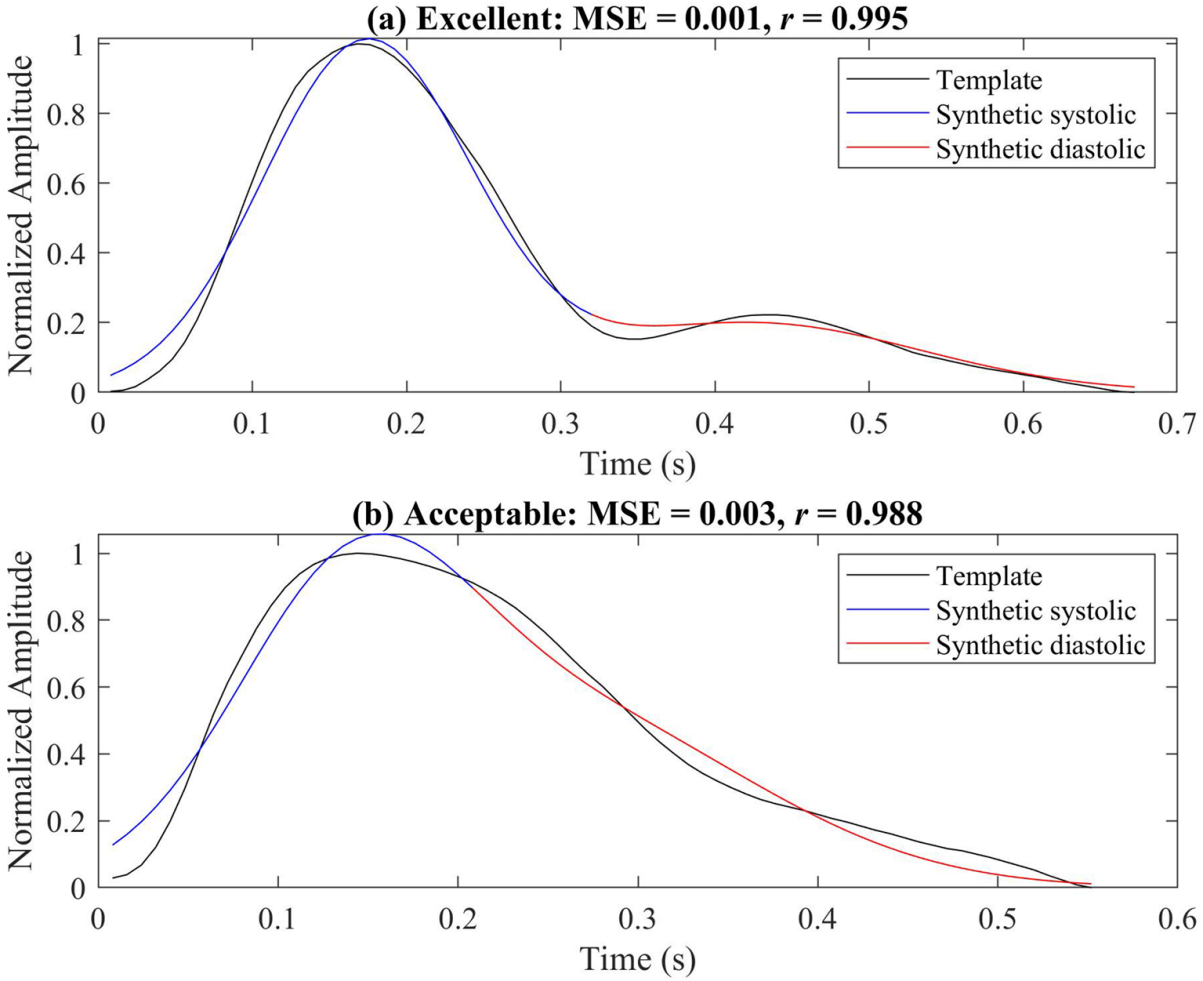
Synthesized PPG signal using (**a**) excellent pulse template and (**b**) acceptable template. The optimal parameters for generating synthesized PPG signals based on these templates are shown in Table 1. An excellent PPG template means that the systolic and diastolic waves are salient (more clinical features, including heart rate, can be determined). An acceptable PPG template means that the systolic and diastolic waves are not salient (only heart rate can be determined). Here, MSE is the mean square error between the synthetic PPG and the template PPG, and *r* is the correlation coefficient. This figure shows that the model can generate excellent and acceptable PPG pulses with high correlation and low MSE.

Figure 2 shows the morphology of the excellent and acceptable template PPG and the synthetic PPG using the optimization parameters. And Fig. 5 shows the result of simulating the unfit template. The corresponding optimized parameters, MSEs, and correlations are shown in Table 1. This model achieved a high correlation and low MSE when simulating the excellent and acceptable templates. Note that the length of these templates was different.

**Table 1:** The optimal parameters for each PPG template obtained using the interior point method.

Template	$a_1$	$\theta _1$	$b_1$	$a_2$	$\theta _2$	$b_2$	MSE	*r*
Excellent	1.0000	− 1.5161	0.6303	0.1999	0.8186	1.0225	0.001	0.995
Acceptable	0.7303	− 1.5510	0.7283	0.5291	− 0.2553	1.2271	0.003	0.988
Unfit	0.9288	− 1.0241	1.2055	0.4916	2.2684	1.2055	0.017	0.851

$\theta _1$ and $\theta _2$ are the position of the peak centers of the first Gaussian function and the second Gaussian function, respectively; $a_1$ and $a_2$ are the peak heights, $b_1$ and $b_2$ are the standard deviations of the first and second Gaussian functions; respectively. MSE is the mean square error, and *r* is the correlation coefficient. An excellent PPG template means that the systolic and diastolic waves in pulse are salient. An acceptable PPG template means that the systolic and diastolic waves are not salient, but heart rate can be determined. Unfit means that the heart rate cannot be determined and the systolic and diastolic waves cannot be distinguished.

### Variability of beat-to-beat intervals

Valley-to-valley intervals are always similar to peak-to-peak intervals in PPGs; both of them correspond to heart rate^20^. To test the pulse duration generated by the proposed model, we compared heart rate variability (HRV) of the synthetic PPG and the real PPG. The real PPGs are the 116 subjects from the MIMIC III database. For each real PPG, we synthesized a PPG by the proposed model with the same mean heart rate and standard deviation of the beat to beat intervals. To look for the peaks, the real PPG is filtered by the Chebyshev II filter at the frequency range 0.5–15 Hz. And then, a simple algorithm that looks for local maxima within a small window was used to detect the peaks. After synthesizing the PPG without noise, the synthesized PPG will undergo the same processing to obtain peaks. These peaks were used to calculate the HRV parameters. Note that the synthetic PPG is the same length as real PPG.

HRV is defined as the variation in the time interval between heartbeats. We compared the HRV parameters of real PPG and synthetic PPG for the following: standard deviation of beat to beat intervals (SDNN) and root mean square of the successive differences between adjacent beat-to-beat intervals (RMSSD) in the time domain, low-frequency (LF) power and high-frequency (HF) power in the frequency domain, the standard deviation of the Poincare plot (PP) perpendicular to the line of identity (SD1) and the standard deviation of the PP along to the line of identity (SD2)^21,22^. LF is the total spectral power of all beat-to-beat intervals between 0.04 and 0.15 Hz, while HF is total spectral power between 0.15 and 0.4 Hz.

**Figure 3:**
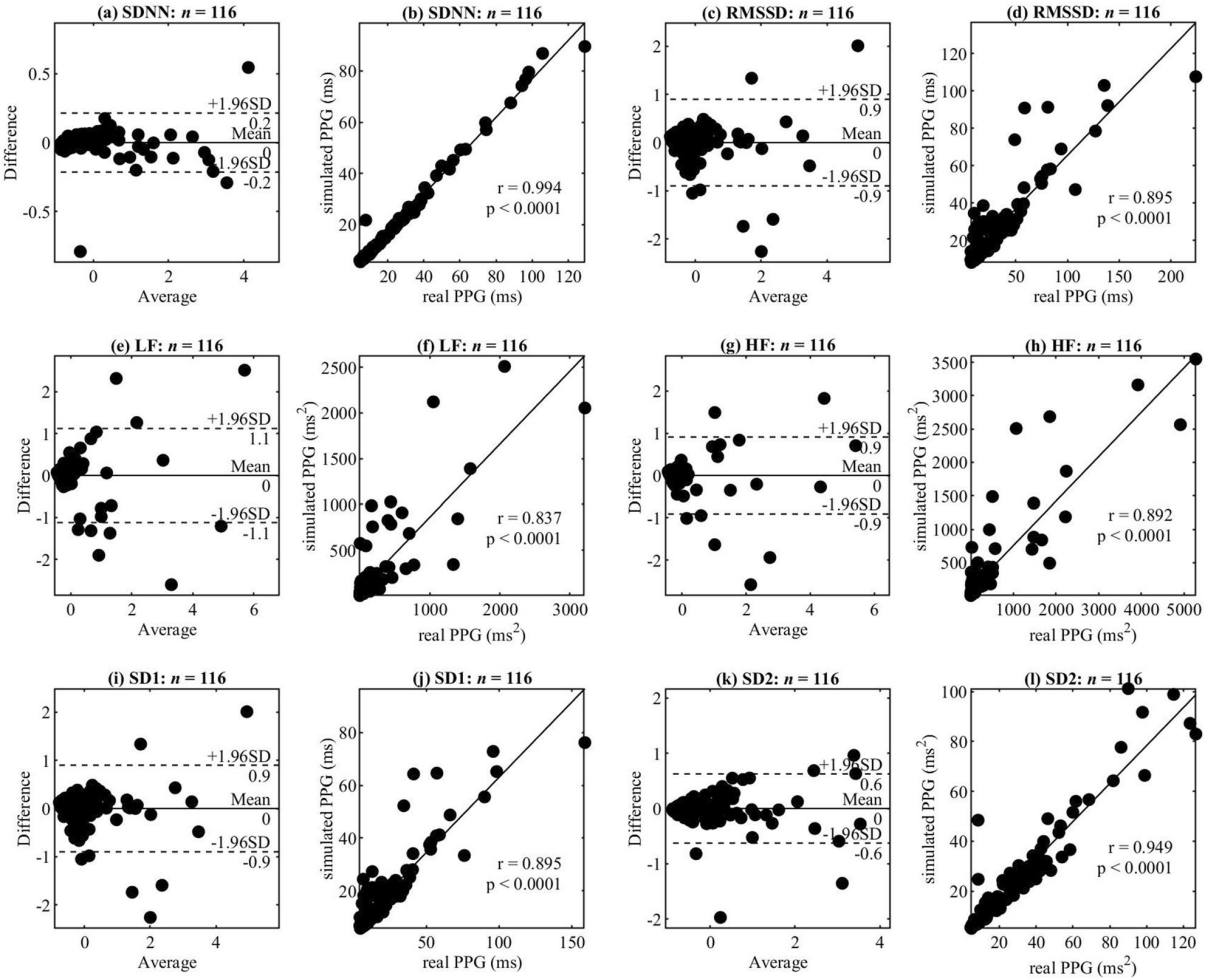
Comparison of HRV between the real PPG and the synthetic PPG over 5-min duration in 116 subjects. (**a**), (**c**), (**e**), (**g**), (**i**) and (**k**) are Normalized Bland-Altman Plots of SDNN, RMSSD, LF, HF, SD1 and SD2, respectively. $$Average = (simulate' + real')/2$$ and $$difference = real' - simulate'$$ where $$real'$$ and $$simulate'$$ are the normalized values of HRV parameters for real PPG and synthetic PPG signals. The equation of normalization is $$X' = (X -$$mean of $$X)$$$$/$$standard deviation of $$X$$, where $$X$$ stands for the HRV parameters. (**b**), (**d**), (**f**), (**h**), (**j**) and (**l**) are correlations of SDNN, RMSSD, LF, HF, SD1 and SD2, respectively. *r* is the correlation coefficient. *p* is the *p* value. ms = milliseconds. This figure shows that the model can obtain a synthetic PPG with a similar HRV to a real PPG by given the same mean heart rate and standard deviation of beat-to-beat intervals.

Figure 3 shows the comparison of the HRV between the synthetic PPG without noise and the real PPG over a 5-min interval of data for the 116 records. Pulse parameters of the synthetic PPG corresponded to those of the excellent pulse template. Figures (a), (c), (e), (g), (i) and (k) are Normalized Bland-Altman Plots of SDNN, RMSSD, LF, HF, SD1, and SD2, respectively. $$Average = (simulate' + real')/2$$ and $$difference = real' - simulate'$$ where $$real'$$ and $$simulate'$$ are the normalized values of HRV parameters for real PPG and synthetic PPG signals. The equation of normalization is $$X' = (X -$$mean of $$X)$$$$/$$standard deviation of $$X$$, where $$X$$ stands for the HRV parameters. Figures (b), (d), (f), (h), (j) and (l) are correlations of SDNN, RMSSD, LF, HF, SD1, and SD2, respectively. The HRV of the synthetic PPG was correlated to the real PPG according to the mean heart rate and SDNN.

### Noise addition

**Figure 4:**
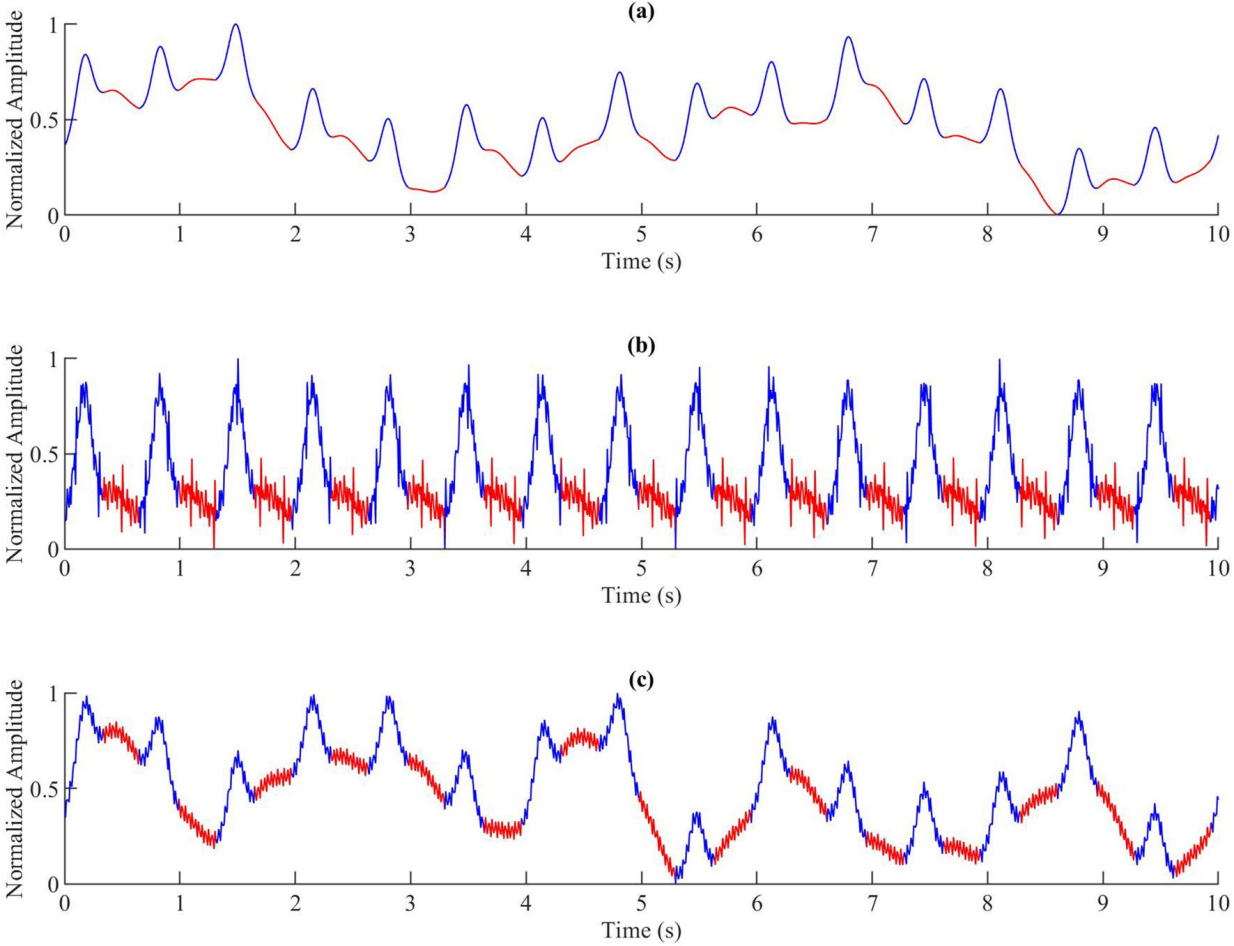
Comparisons of synthetic PPGs with different noises. (**a**) Three different low-frequency noise levels were added. The amplitudes of noise signals are 0.3, 0.4 and 0.1, and the frequencies are 0.3 Hz, 0.2 Hz and 0.9 Hz, respectively.(**b**) Five different high-frequency noise signals were added. The amplitudes of noise signals are 0.1, 0.03, 0.05, 0.04 and 0.05, and the frequencies are 50 Hz, 60 Hz, 70 Hz, 90 Hz and 100 Hz, respectively. (**c**) Five different noise signals were added. The amplitudes of noise signals are 0.4, 0.2, 0.3, 0.02 and 0.04, and the frequencies are 0.1 Hz, 0.7 Hz, 0.5 Hz, 70 Hz and 90 Hz, respectively. The blue part in (**b**) is the systolic wave and the red part is the diastolic wave. This figure shows that the synthetic PPG generated by the proposed model is similar to the real PPG in the MIMIC III database.

Figure 4 shows the 10-s synthetic PPG generated by the dynamic model. Different levels of noise were added to Fig. 4a–c. Min-max normalization was performed after adding noise. Three different values of low-frequency noise were added in Fig. 4a, five different values of high-frequency noise were added in Fig. 4b, and a mixture of five different values, including low and high-frequency noise, was added in Fig. 4c.

## Discussion

This work developed a dynamic model for generating synthetic PPGs with any sampling frequency and duration that are characteristic of real PPGs. The average morphology can be controlled by specific parameters as in dynamic ECG models^23^. Table 2 compares our work to other studies. The reason behind using only two Gaussian functions to represent the main two phases of the cardiac cycle: Diastole and systole. Increasing the number of Gaussian functions can improve the fit accuracy^16^, but the advantage of using only two Gaussian functions is that the function parameters correspond to the features of the systolic and diastolic peaks. We used MSE and correlation as similarity measures to compare between the original PPG signal and synthesized. The reason behind using MSE is it is a very popular distance measure for quantifying similarity^24^, and the reason behind using Pearson’s Correlation coefficient is the robustness in quantifying morphological changes in time series physiological signals such as PPG^25^. To obtain optimal parameters for the model, the two similarity measures were used simultaneously. In other words, we optimized the model’s parameters based on MSE and Pearson’s correlation coefficient at the same time. By using the optimized parameters, this model can simulate both excellent and acceptable real PPG pulses even if only two Gaussian functions are used; however, it is not highly correlated for unfit templates. It is difficult to accurately extract the features using the morphology of unfit PPG beats. If a higher fit accuracy is required, we could easily add more Gaussian functions in a single pulse to the model.

**Table 2:** Comparison of different published studies.

Year	Author	# of functions	Number of parameters	Signal dependent	Signal length	Beat-to-beat variability	HRV validation
2020	This work	2 Gaussians	6	None	Dynamic	Yes	Yes
2017	Solosenko et al.^18^	1 log-normal and 2 Gaussians	12	ECG	Dynamic	No	N/R
2014	Martin-Martinez et al.^17^	2 Gaussians	10	Original PPG	Dynamic	Yes	N/R
2013	Wang et al.^16^	4 or 5 Gaussians	12 or 15	None	Fixed	No	N/R

This table shows that the proposed model uses only two Gaussian functions with six parameters to generate random synthetic PPG with different lengths, without the need for additional biosignals. HRV is heart rate variability and N/R means not reported.

The dynamic model converts the independent variable *t* in the Gaussian functions from time to angle using the *arctan*2 function. The six model parameters are then used as is without additional tuning for generating PPG waveforms. Optimized model parameters (obtained from tuning a certain PPG template) can be used for synthesizing modified forms of the PPG signal by changing the sampling rate, morphology and duration. This formulation could be considered as an improvement over previous formulations. Moreover, the formulation of the model is more clinically appealing and interpretable as the two Gaussian functions are representing systole and diastole.

Another advantage of the proposed model is that it can generate PPG signals without using additional biosignals such as ECG. The proposed model generates beat-to-beat intervals in a random fashion to simulate real PPG signals. Martin-Martinez^17^ used two autoregressive moving average models to approximate beat-to-beat intervals based on real PPG signals; however, it requires the original PPG to generate a synthesized PPG signal. Sološenko^18^ used an additional biosignal (from an ECG) to induce variability in the beat-to-beat durations of the synthesized PPG signal. In contrast, our proposed model only requires the mean HR and the standard deviation of the beat-to-beat intervals to synthesize a PPG without the need for additional biosignals.

**Figure 5:**
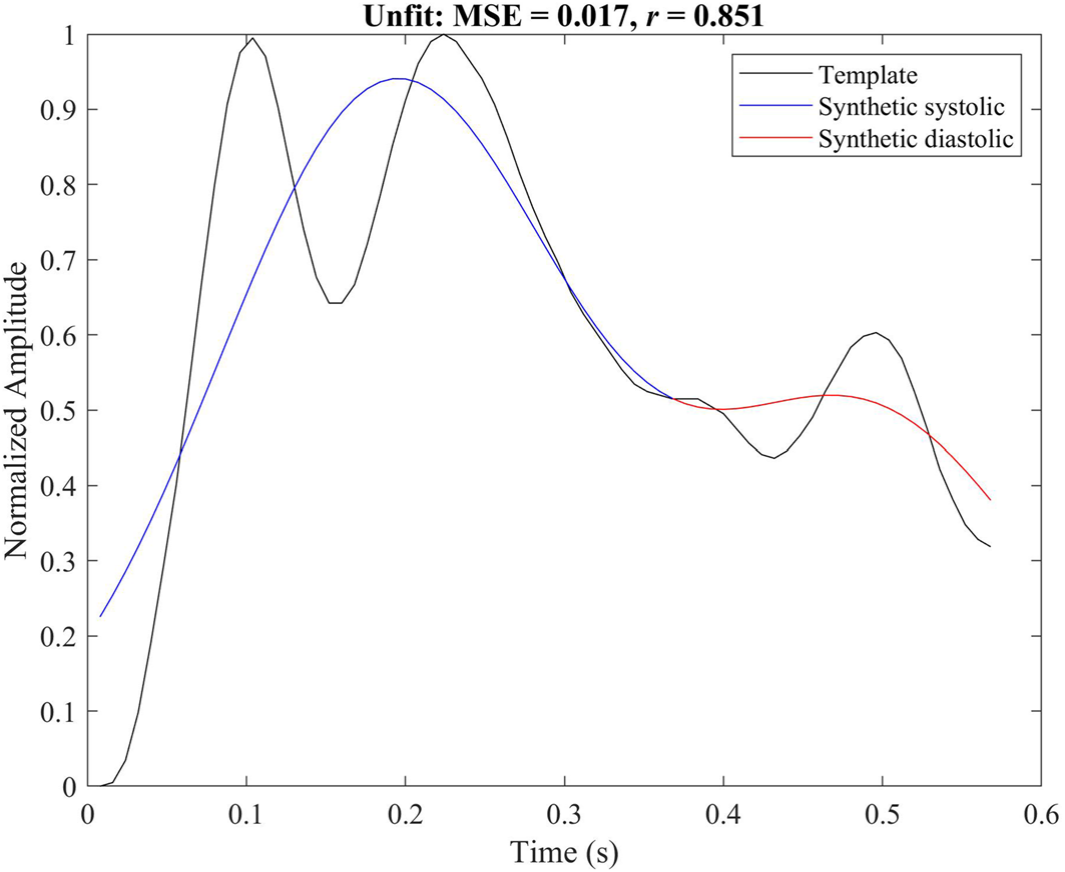
Synthesized PPG waveform based on the unfit template. The optimal parameters of the synthesized unfit PPG waveform are shown in the row corresponding to the unfit group in Table 1. Unfit means that the heart rate cannot be determined and the systolic and diastolic waves cannot be distinguished. Here, MSE is the mean square error between the synthetic PPG and the template PPG, and *r* is the correlation coefficient. This figure shows that the proposed model cannot synthesize the unfit PPG pulses.

The objective of this work was to define a model that could generate synthetic PPGs representing excellent and acceptable PPG waveforms. The model could not simulate unfit forms. As shown in Fig. 5, the MSE between the synthetic PPG and the real PPG was 0.0166 and the correlation was 0.85 for the unfit template. The optimization parameters are shown in Table 1. The optimization algorithm stopped when the current step size was less than the selected value of the step-size tolerance $(1\times 10^{-10})$. The value of the objective function cannot be close to zero when the PPG is unfit.

Using two Gaussian functions to represent a single PPG waveform is not sufficient to adequately capture the morphological changes of the unfit template. Therefore, one of our next steps is to increase the number of Gaussian functions to generate different PPG morphologies, including unfit waveforms. Note that the beauty of the proposed method is the clinical interpretability of the model as the Gaussian functions are representing the systolic and diastolic waves in PPG waveform. The increase in Gaussian functions could be less clinically meaningful.

Another limitation of this work is that arrhythmia is not considered due to the lack of clinical information available for arrhythmic subjects. It is known that arrhythmia causes changes in the duration and amplitude of the PPG pulse^18^. These changes impact the morphology of arrhythmia PPG waveforms different from that of normal ones. We plan to use other databases to determine the effect of different types of PPG waveform morphologies, and induce arrhythmic beats into the normal synthetic signal. We will also test event detectors (e.g., to detect systolic waves) based on this model. However, the proposed model could allow us to understand the underlying changes in abnormalities (e.g., changes of parameters for generating systolic and diastolic waves in subjects with and without hypertension).

It is essential to have the ability to add different types of noise to the simulated PPG signals to simulate different real-life conditions. For example, in the case of signal-to-noise ratio greater than 2, adding low-frequency noises (< 0.5 Hz) may cause a baseline wander, adding the noises in which the frequency is between 0.5 and 15 Hz may change the morphology of PPG pulse. High-frequency noises (greater than 30 Hz) may make the signal not smooth. Dark noise or white noise will have the above effects at the same time due to the wide spectrum. We can add different types of noise according to different needs.

## Conclusions

The dynamic model presented in this study, based on two Gaussian functions, offers a promising new method for analyzing and synthesizing PPG signals. The synthesized PPG signal can be generated with different beat-to-beat intervals, similar to a real PPG signal. The model can better simulate the characteristics of the excellent and the acceptable PPG templates with a fewer number of parameters when compared with currently available models. Further research is needed to generate arrhythmic PPG signals.

## Methods

The PPG generator consists of two main parts: modeling a single PPG waveform and generating a PPG signal. The main idea behind the work is to generate a sequence of PPG waveforms based on the circular motion principle, as shown in Fig. 1.

### Modeling single PPG pulses

**Figure 6:**
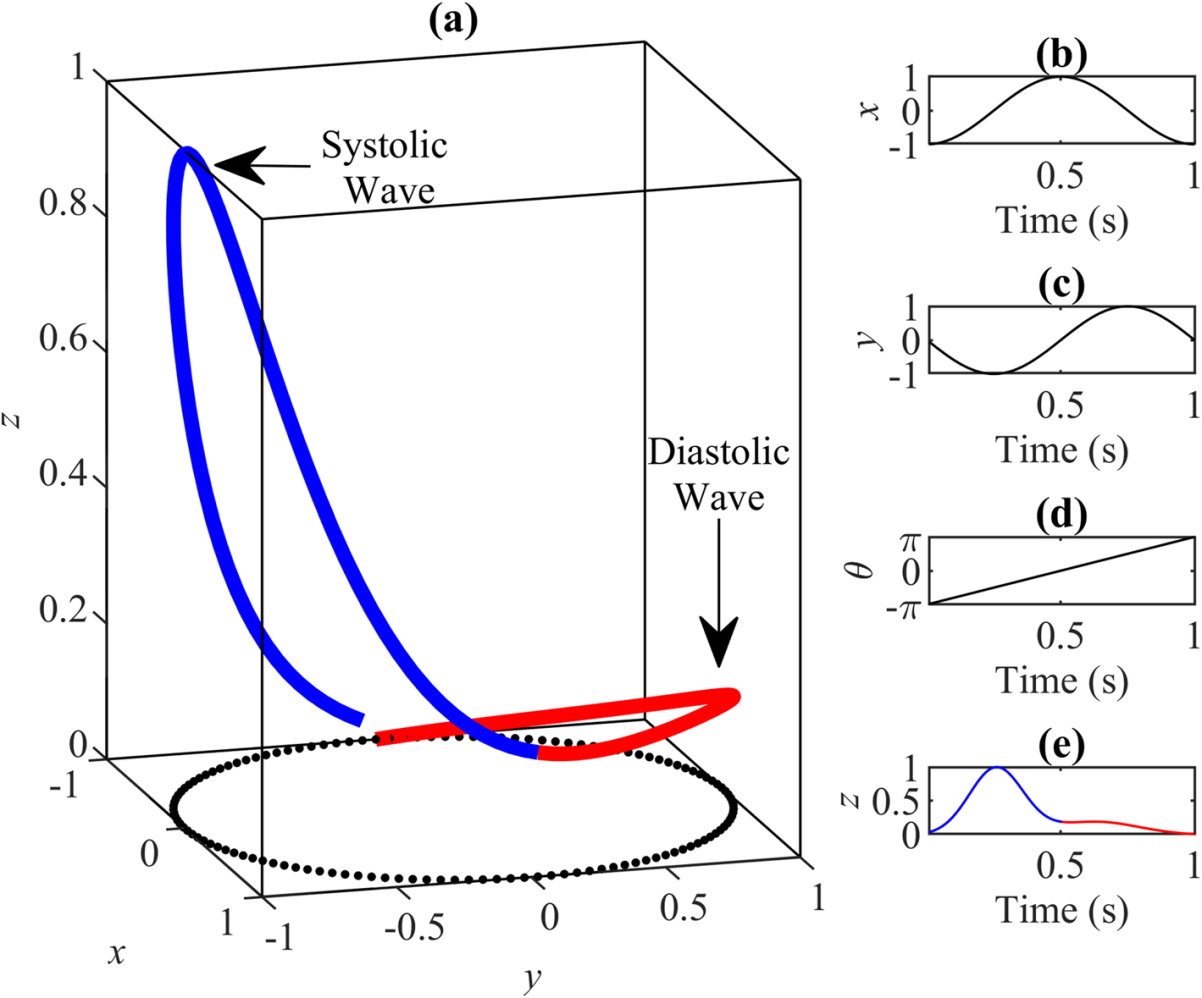
Motion trajectory of a single synthesized PPG waveform. The simulated PPG waveform is based on an excellent PPG template (associated model parameters are shown in Table 1). The blue curve represents the first Gaussian, which corresponds to the systolic wave, and the red curve represents the second Gaussian, which corresponds to the diastolic wave. (**a**) Synthesized PPG waveform trajectory of one heartbeat in 3-D space, and the dotted part is the unit cycle. (**b**) Changes to the value of $x$ in one period. (**c**) Changes to the value of $y$ in one period. (**d**) Changes to the value of $\theta$ in one period. (**e**) Changes to the value of $z$ in one period. This figure shows how the model generates a systolic wave followed by a diastolic wave using two Gaussian functions.

In this model, the PPG waveform is the trajectory of motion in the three-dimensional space described using a Cartesian coordinate system (*x*, *y*, *z*). The periodicity of PPG is represented by circular motion, as shown in Fig. 6a. The trajectory of motion in the (*x*, *y*) plane is mapped to the unit circle. One sweep of the circle corresponding to a peak-to-peak interval or heartbeat. (*x*, *y*) is defined as:

2 $$\begin{aligned} {\left\{ \begin{array}{ll} x(t) = \cos ({\omega t})\\ y(t) = \sin ({\omega t}), \end{array}\right. } \end{aligned}$$

where $t$ is the time, and $\omega$ is the angular velocity used to control the duration of the pulse, calculated by:

3 $$\begin{aligned} \omega = \frac{2\pi }{T}, \end{aligned}$$

where $T$ is the duration of the PPG pulse.

The trajectory in the *z* direction is the resulting PPG signal. $\theta$ is introduced as an independent variable for motion in the *z* direction. $\theta$ is the four-quadrant inverse tangent of (*x*, *y*), defined as:

4 $$\begin{aligned} \theta (t) = atan2(y(t),x(t)) \end{aligned}$$

Regardless of the changes to (*x*, *y*), $\theta$ is considered to the range of $(-\,\pi , \pi )$. Peaks on the PPG, such as the systolic peak and the diastolic peak, were simulated by Gaussian functions, defined as:

5 $$\begin{aligned} g(\theta (t)) = A\exp {\left(-\frac{(\theta (t)-\mu )^2}{2\sigma ^2}\right)}, \end{aligned}$$

where *A* is the peak height, $\mu$ is the position of the peak center, and $\sigma$ is the standard deviation of the two Gaussian functions.

These two peaks were placed along the unit circle at fixed angles $\theta _1$ and $\theta _2$. In this model, *z* is the sum of the Gaussian functions for the variable $\theta$. A periodical PPG waveform is generated through circular motion as follows:

6 $$\begin{aligned} {\left\{ \begin{array}{ll} x(t) = \cos {(\omega (t - t_0) - \pi )}\\ y(t) = \sin {(\omega (t - t_0) - \pi )}\\ z(t) = \sum _{i=1}^2{a_i\exp {\left(-\frac{(\theta (t)-\theta _i(t))^2}{2b_i ^2}\right)}}, \end{array}\right. } \end{aligned}$$

where $t_0$ is the end time of the previous beat, $\pi$ is used to align the initial point of this model to the position of the onset $(\theta = - \pi)$ in a PPG waveform.

The method used for the selection of parameters is introduced in the next section. The corresponding changes to $x$, $y$, $\theta$, and $z$ over a single period are shown in Fig. 6b–e; these are repeated in the next pulses. In this figure, the pulse duration was one second, and the sample frequency was 125 Hz.

### Parametric optimization

The peak height, position of the peak center, and standard deviation of the two Gaussian functions were used to determine the morphology of the synthetic PPG. In this study, three types of real PPG pulse templates (described earlier) were used to evaluate the model. There was one PPG pulse in each type of template. The objective of the optimization step was to determine model parameters that would result in matching the synthetic PPG to the real PPG as closely as possible. The corresponding objective function was expressed as follows:

7 $$\begin{aligned} \begin{aligned} p^{*}=\arg \min _p ( (1 - corr(z_p(n),s(n))) + \sum _{n=1}^l{(z_p(n) - s(n))^2}) \end{aligned} \end{aligned}$$

s.t. $p=\{a_1, \theta _1, b_1, a_2, \theta _2, b_2 \}$. with the constraints:

8 $$\begin{aligned} {\left\{ \begin{array}{ll} 0 \le a_2< a_1 \le 1 \\ 0 \le b_1< b_2 \le 2\\ -\pi \le \theta _1 < \theta _2 \le \pi ,\\ \end{array}\right. } \end{aligned}$$

where $z_p(n)$ is the synthetic PPG, $l$ is the length of the real PPG $s(n)$, and $corr$ is Pearson’s linear correlation coefficient, to test the correlation between the synthetic PPG and the real PPG, which in turn, calculated as follows:

9 $$\begin{aligned} corr(\alpha ,\beta ) = \frac{\sum _{i=1}^n{(\alpha _i - {\overline{\alpha }})(\beta _i - {\overline{\beta }})}}{\sqrt{\sum _{i=1}^n{(\alpha _i-{\overline{\alpha }})^2}}\sqrt{\sum _{i=1}^n{(\beta _i-{\overline{\beta }})^2}}}, \end{aligned}$$

where *n* is the signal length. $\alpha _i$, $\beta _i$ are the individual points with index *i*, and ${\overline{\alpha }}$, ${\overline{\beta }}$ are the mean value of $\alpha$, $\beta$. In this study, the interior-point method^26^ was used to solve the optimization problem.

### Pulse duration generator

It is clear from the model that the pulse durations are equal to the onset-to-onset intervals, also called valley-to-valley intervals in synthetic PPG signals. The difference between valley-to-valley intervals is represented by the angular velocity $\omega$. The dynamic model can repeat the morphology to generate the required signal length. By providing a series of valley-to-valley intervals, the model can synthesize a continuous PPG.

The pulse durations in the synthetic PPG are generated using the normal distribution random function with mean and standard deviation. The mean value and the standard deviation are calculated according to the mean heart rate and the standard deviation of beat-to-beat intervals provided by the user. Variability of parameters on a beat-to-beat basis was not considered in this study.

### Noise addition

After synthesizing the clean signal, the noise was incorporated into the signal based on the specific need identified. In this work, a simple way to incorporate noise signal was introduced. The noise signal was defined as:

10 $$\begin{aligned} \delta (t) = B\sin {2\pi ft}, \end{aligned}$$

where $B$ is the amplitude of the noise, and $f$ is the noise frequency. For instance, an example of incorporating low-frequency noise, such as baseline wander, would be to adopt an amplitude of 0.4 and frequency of 0.2 Hz. Another example of incorporating high-frequency noise would be to adopt an amplitude of 0.02 and frequency of 50 Hz. If necessary, one can add a combination of different frequencies and amplitudes, even including different types of noise to the same synthesized PPG signal.
